# Phonological and orthographic cues enhance the processing of inflectional morphology. ERP evidence from L1 and L2 French

**DOI:** 10.3389/fpsyg.2014.00888

**Published:** 2014-08-13

**Authors:** Haydee Carrasco-Ortiz, Cheryl Frenck-Mestre

**Affiliations:** ^1^Universidad Autónoma de QuerétaroSantiago de Querétaro, Mexico; ^2^Laboratoire Parole et Langage, Centre National de Recherche ScientifiqueAix-en-Provence, France; ^3^Aix-Marseille UniversitéAix-en-Provence, France

**Keywords:** ERPs, verbal inflection, late bilinguals, phonological processing, sentence processing

## Abstract

We report the results of two event-related potential (ERP) experiments in which Spanish learners of French and native French controls show graded sensitivity to verbal inflectional errors as a function of the presence of orthographic and/or phonological cues when reading silently in French. In both experiments, verbal agreement was manipulated in sentential context such that subject verb agreement was either correct, ill-formed and orally realized, involving both orthographic and phonological cues, or ill-formed and silent which involved only orthographic cues. The results of both experiments revealed more robust ERP responses to orally realized than to silent inflectional errors. This was true for L2 learners as well as native controls, although the effect in the learner group was reduced in comparison to the native group. In addition, the combined influence of phonological and orthographic cues led to the largest differences between syntactic/phonological conditions. Overall, the results suggest that the presence of phonological cues may enhance L2 readers’ sensitivity to morphology but that such may appear in L2 processing only when sufficient proficiency is attained. Moreover, both orthographic and phonological cues are used when available.

## INTRODUCTION

Can one reed a book? Indeed, whether or not one necessarily activates phonological representations when reading and accessing the meaning of a word is a long standing debate in reading research (McCusker et al., 1981; Morris and Folk, 2000; Harm and Seidenberg, 2004). Early theories assumed that phonological recoding was a secondary, slower, route to meaning as compared to “direct access” via orthographic codes alone (Paap and Noel, 1991). This view has been seriously challenged in the last 15 years. Today’s debate lies not in the question of whether phonological information is retrieved but how and when, i.e., whether phonological information is retrieved pre-lexically or only once a stored lexical form has been activated on the basis of orthography, thus giving rise to stored phonological information (for recent reviews see Van Orden and Kloos, 2005; Hino et al., 2013).

The strongest evidence for phonological mediation during the processing of written words has been provided by research on single-word reading (Van Orden, 1987; Lukatela and Turvey, 1993; Jared et al., 1999). Seminal behavioral work in this area has shown that the activation of phonological information is both rapid and automatic, while perhaps lagging one beat behind that of orthography (Perfetti and Bell, 1991; Ferrand and Grainger, 1993). Said findings have since been replicated using event-related potentials (ERPs; Grainger et al., 2006).

Less attention has been paid to the role of phonological information during sentence processing; however, those studies that have examined this question have shown benefits. In both English and in French, a “preview” of the phonological information contained in the upcoming word in the sentence facilitates the reading of said word (Pollatsek et al., 1992; Rayner et al., 1995, 1998; Miellet and Sparrow, 2004; Ashby et al., 2006; but see Daneman and Reingold, 2000). The effect is not restricted to alphabetic languages; it has also been reported for logographic languages, such as Chinese (Pollatsek et al., 2000; Liu et al., 2002; Tsai et al., 2004), The finding that the prior activation of phonological information enhances reading performance, whichever the language under question, adds considerable weight to the hypothesis that such is part and parcel of the natural reading process (although see Van Orden and Kloos, 2005; Hino et al., 2013 for further discussions of these phonological effects).

The present study examined the question of phonological recoding, but from a syntactic viewpoint. We examined the impact of phonological cues on the processing of subject–verb agreement in sentential context. Moreover, we examined this question in both first (L1) and second language (L2) processing. Indeed, this issue is important not only for understanding the contribution of phonology to morphological processing in native readers but also in understanding whether phonology can be useful in learning verbal inflection in an L2. The language under question was French, which presents a particularly interesting case to study how phonological representations may influence how the orthographic code is processed.

Processing written French requires dealing with numerous morphological inflections that do not have overt phonological representations. Regular inflections of the present tense are an illustrative example of this phonetic opacity. Morphological variations are not systematically represented in the phonological code; verbal inflections of the three singular persons for regular (first group) verbs are phonologically identical, despite the orthographic variation for the second singular person [e.g. *tu parles* 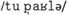 “you speak” is pronounced the same as *je/il parle* 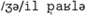 “I/he eat(s)”]. Analogously, the third person plural inflected form is phonologically identical to that of the three singular persons regardless of orthographic variation across forms [e.g. *ils parlent* 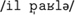 “they speak” is pronounced the same as *je/tu/il parle(s)* “I/you/he speak(s)”]. The absence of audible distinctions for different morphological forms in written French seems to account for the majority of errors made in verbal agreement production (Brissaud and Sandon, 1999; Negro and Chanquoy, 2000; Largy and Fayol, 2001; Chevrot et al., 2003). These studies have indeed shown that the presence of an audible morphological marker considerably reduces verbal agreement errors in children and skilled literate adults alike. Akin to the results obtained for the French language, both Dutch children and experienced adults produce more spelling errors on regularly inflected verb forms that do not include audible differences than for those that do (Frisson and Sandra, 2002; Sandra et al., 2004).

The effect of phonological mediation on inflected morphology can also be seen in comprehension, although debate indeed remains as to the systematic nature of this effect. In a self paced reading study, Brysbaert et al. (2000) examined whether orthographic information is necessary and/or sufficient to process homophonous verb forms and how the reading system deals with this type of silent morphological information. To do so, they looked at the respective contribution of orthographic and phonological cues on the processing of verbal tense in short sentence contexts. Results for native Dutch readers showed that orthographic cues alone sufficed to process the tense of the verb. These results were interpreted as evidence that the reading system is sensitive to orthographic information that is not represented at the phonological level. However, according to the authors, these findings could not be taken as evidence against the use of phonological cues in normal reading. Indeed, the simple presence of homophonic verb forms may have triggered the alternative use of orthographic information in order to disambiguate the verb. In line with the model forwarded by Harm and Seidenberg (2004), numerous empirical studies have shown that orthographic and phonological information can be activated in parallel during silent reading (cf. Van Orden and Kloos, 2005, for a review).

Studies that used ERPs to investigate the processing of inflectional morphology during written sentence processing have provided further understanding about the role of phonological cues in this process, in both native and non-native speakers (Osterhout et al., 2004, 2006; Frenck-Mestre et al., 2008). In a longitudinal study, Osterhout et al. (2004) suggested that L2 learners can quickly integrate morphosyntactic features with minimal instruction (e.g. 4 months), especially when features are present in the native language and morphological inflections involve phonological markers. Results showed that English L1–French L2 learners elicited an ERP effect in response to verbal agreement violations that presented phonological cues [e.g.Tu adores\*adorez “You adore(s)”], whereas no variation in the ERP responses was observed in response to determiner-noun agreement errors, which were both largely absent from the native grammar and that involved silent morphemes [e.g. Tu manges des hamburgers\*hamburger “You eat hamburger(s)”]. However, it remained unclear whether it was the similarity of grammatical features across L1–L2 alone, or this factor combined with the oral realization of morphemes that lead to faster learning and a more systematic brain response to violations.

To isolate the effects of phonological realization on morphosyntactic processing, subsequent studies manipulated the presence versus the absence of phonological cues to grammatical morphemes for shared grammatical features in L1 and L2 (Frenck-Mestre et al., 2008; McLaughlin et al., 2010). Frenck-Mestre et al. (2008) examined the impact of phonological realization on verbal agreement in both native and non-native speakers of French. Results showed that compared to grammatically correct sentences, verbal agreement errors involving orally realized morphemes elicited a P600 effect for both native French speakers and German L1–French L2 learners. In contrast, silent inflectional errors produced different ERP patterns across the two groups. French native speakers showed a smaller P600 effect in comparison to orally realized errors whereas for L2 learners no robust effects were found in response to silent errors. These authors concluded that the presence of phonological information facilitates the processing of verbal agreement errors in native speakers and enhances the learning rate of verbal inflection in L2 learners of French.

The effects of phonological realization on morphosyntactic learning were also examined longitudinally in non-native speakers by McLaughlin et al. (2010). ERPs were recorded in three consecutive sessions while English L1–French L2 learners read the same materials as those used by Frenck-Mestre et al. (2008). At the end of the third session, a subgroup of learners presented an N400 effect to verbal agreement violations. By contrast, the other subgroup of learners, who first elicited an N400 response to inflectional errors, showed a subsequent small P600 effect in this third session. Although ERP differences were not observed as a function of whether the inflectional errors were phonologically realized or silent, learners’ acceptability judgments were indeed sensitive to the presence of phonological cues. According to the authors, the processing of orthographic cues may not have triggered the activation of phonological information in L2 learners in contrast to the results found for native speakers and more proficient learners (Frenck-Mestre et al., 2008). However, the presence of oral cues does nonetheless seem to have an effect on morphological learning in the early stages of acquisition as evidenced by the learners’ behavioral responses.

The current set of studies further investigated the extent to which phonological cues impact upon the processing of inflectional morphology in silent reading. Thus far, behavioral and ERP studies have presented inconsistent results with respect to the specific contribution of phonological cues during morphosyntactic processing in both native and non-native language readers (Brysbaert et al., 2000; Frenck-Mestre et al., 2008; McLaughlin et al., 2010). Indeed, different levels of sensitivity to phonological cues have been suggested in the literature discussed above. Thus, in two experiments, we aimed to determine the extent to which native and non-native readers are sensitive to the presence of phonological cues when processing inflectional morphology. To do so, we recorded ERPs from native French speakers as well as from Spanish L1–French L2 learners while they read sentences that contained subject–verb agreement errors which were either phonologically realized, i.e., when morphological information involved both orthographic and phonological cues, or silent, i.e., when morphological information involved orthographic cues that were not orally realized (cf. **Table 1**).

**Table 1:** Examples of the three sentence conditions (correct, incorrect and phonologically realized, incorrect and silent) for the six different verbal persons in French.

Sentence onset	Correct	Incorrect, phonologically realised	Incorrect, phonologically silent	Sentence end
Le matin	je mange 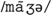 tu manges il/elle mange nous mangeons vous mangez ils/elles mangent	mangez 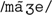 mangez mangez mangent 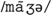 manges 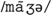 mangeons 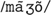	manges mange manges manges	du pain

In a first experiment, we wished to determine whether the absence of an effect of phonological cues to verbal morphology in L2 learners reported by McLaughlin et al. (2010) was indeed attributable to the relatively low proficiency of these learners. If phonological recoding only comes into play at a more advanced level of L2 proficiency, we should find that more proficient L2 learners show the effect. To address this question, we examined L1 Spanish speakers who were immersed in the French language and who had several years of formal instruction in this language. Based on the findings reported by Frenck-Mestre et al. (2008), we predicted that these more advanced learners would show sensitivity both online and oﬄine to verbal inflected violations and that such would vary as a function of the presence of phonological cues.

In a second experiment, we examined whether the presence of orthographic cues in addition to phonological ones may enhance processing of inflectional morphology. Brysbaert et al. (2000) found that the added presence of phonological cues did not allow participants to recover the tense of verbs faster, as compared to both orthographically and phonologically ambiguous forms. They concluded that readers can process verbal inflection as easily when silent as when orally realized. To address this issue, in our second experiment we manipulated verbal inflections such that orthographic variation was held constant across varying phonological conditions. Both a new control group of native French speakers and a new group of advanced Spanish–French bilinguals were tested.

The two groups of Spanish–French late bilinguals recruited for the present study were considerably more advanced than the English–French late L2 learners who participated in the McLaughlin et al. (2010) study. Importantly, all of our L2 participants were enrolled in university level classes conducted in French at a French university and had been immersed in the French language for roughly 6 months at the time of the study. There is clear evidence that direct classroom instruction on phonological form can improve learning rate of French grammar (Arteaga et al., 2003). Nonetheless, as highlighted by Muñoz (2008, 2014), we can assume in line with the data from numerous linguistic and psycholinguistic studies that immersion will exert an important influence on L2 proficiency. As concerns the ability to compute syntactic structures, late L2 learners who have been immersed in the L2 for several years show a preference for resolving ambiguous structures in a manner similar to native speakers, quite unlike less experienced L2 learners who show an influence of their L1 (Frenck-Mestre, 2002; see also Dussias, 2003). It is important to underline that such a result is not a product of any explicit training but simply the by-product of extended experience with the L2 in daily life. More recently, Pliatsikas and Marinis (2013) reported successful online, immediate processing of complex syntactic structures (filler-gap dependencies) in a group of L2 English speakers who had been immersed for several years in their L2 environment but not for L2 learners with less exposure. Again, no specific training on said structures was given; with more years of experience, the late L2 learners simply achieve a high level of automatized syntactic parsing. Note, these findings are at odds with the claim that late L2 learners make do with shallow parsing based on heuristics (Clahsen and Felser, 2006), but argue in favor of models which assume that provided sufficient experience with/exposure to a language adult learners will achieve “nativelike” syntactic parsing (Herschensohn, 2000; Hopp, 2010; Steinhauer, 2014).

There is also some electrophysiological evidence that adult learners benefit from “naturalistic” or implicit learning as opposed to explicit instruction when confronted with a new language. Morgan-Short et al. (2012) reported that adult learners who were given implicit instruction on an artificial grammar, somewhat akin to what happens in immersion, showed “more native like” cortical responses to violations of word order than did a group of learners with equal exposure but explicit instruction. The pattern of ERP results was nonetheless complex. Participants were tested twice, once when at a low level of proficiency in the artificial language and again, following more training when they had achieved a high level of proficiency. Morgan-Short et al. (2012) reported that at higher proficiency, the implicit learning group showed a “native-like” pattern, consisting of an anterior-negativity followed by a P600 response to word order violations, whereas the explicit learning group showed only a P600. First, as noted by many and recently reiterated by Steinhauer (2014) and Tanner (2014), the presence of an early negativity (whether left lateralized or not) in response to syntactic processing difficulty is not systematic enough to be considered the hallmark of automatic syntactic parsing, whether in a native or late learned language (but see Molinaro et al., 2011). Perhaps more convincingly, at the lower level of proficiency the implicit learning group showed an N400 effect (albeit preceded by an early negativity, from 150 to 300 ms and extending to 700 ms), which then became a P600 (albeit preceded by the same early negativity – which may simply have been the remnant of the N400) at the second testing period. The explicit training group showed no ERP response when at a low level of proficiency. Hence, if nothing else, the implicit learning elicited a cortical response earlier than did explicit training. It is of interest that this is the first study, to our knowledge, to show an “N400–P600” transition for aurally presented sentences, and for artificial grammars.

In both experiments, we expected the cortical response to grammatical violations to vary as a function of the presence of overt phonetic cues to grammatical morphemes. Exactly what ERP signature we could expect to show variation depends upon the particular study that one considers. In Frenck-Mestre et al. (2008) variation in the P600 response was found in both native and L2 speakers but of different types; in native speakers, orally realized inflectional errors produced a larger P600 response than silent errors. In L2 speakers, orally realized errors elicited a significant P600 response while silent errors produced only a hint of variation and in the N400 response. Seminal work by Osterhout et al. (2004) has shown that adult L2 learners can show either the more typical P600 response or an N400 effect when confronted with violations of inflectional morphology, and that some L2 learners show a gradual shift from an N400 effect to a more typical P600 response as a function of grammatical competence. This result has since been replicated, both in late L2 learners (McLaughlin et al., 2010; Tanner et al., 2013) and in “heritage” L2 speakers, i.e., speakers whose parents are native speakers but who were raised and schooled in an environment where the ambient language was not that of their parents (Tanner et al., 2013). Other L2 studies have also reported N400 effects in response to grammatical violations, but as a function of grammatical structure rather than competence, with the same L2 speakers showing either an N400 or a P600 depending upon the familiarity with/frequency of structures in the L2 (Foucart and Frenck-Mestre, 2012). It is of importance to note that an N400 effect to syntactic violations is not specific to L2 processing. Osterhout (1997) clearly demonstrated that native speakers can show an N400 effect rather than a P600 in response to violations of syntactic expectancies, especially if the critical word is sentence final. This result, although often swept under the rug in L1 studies of syntactic processing where a P600 dominates in the averaged waveform, especially if the critical word is sentence medial, is well known to any who have looked at their individual data (see also Osterhout et al., 2004). This issue has recently been revisited by Tanner and Van Hell (2014). It has also been shown that the magnitude of the P600 response in native speakers is highly linked to their linguistic proficiency (Pakulak and Neville, 2010). Given all of the above, it is plausible to assume that we might find variation in either of these components as a function of the well formedness of our sentence materials and the overt phonological realization thereof. The debate about what these cortical signatures reveal about sentential processing will be presented in the general discussion and in light of the results obtained here (for discussions, see Steinhauer et al., 2009; Molinaro et al., 2011; Tanner, 2014).

## EXPERIMENT 1

The goal of Experiment 1 was to examine the extent to which advanced Spanish L1–French L2 learners rely on phonological cues to process inflectional morphology online. In this aim, we examined the ERP responses of participants while they read sentences that contained either phonologically realized or silent subject–verb agreement errors. If phonological cues are indeed available online to advanced L2 learners, we should replicate previous results (Frenck-Mestre et al., 2008) showing that these cues increase L2 readers’ sensitivity to morphological violations in like fashion to native controls. If phonological cues are not, however, taken into account as systematically during online L2 processing we can predict oﬄine differences for silent and orally realized errors but not variations in the ERP response for the L2 group. Indeed, the question remains open whether the lack of an online effect of phonological cues in the L2 reported by McLaughlin et al. (2010) is attributable to the lower proficiency of the L2 learners as compared to those studied by Frenck-Mestre et al. (2008) or to a generally less systematic use of these cues during L2 processing. To ascertain whether the pattern for our late Spanish–French bilinguals was similar to that previously reported for native French speakers, we report data for a control group, which largely overlapped with that reported in Frenck-Mestre et al. (2008).

### METHOD

#### Participants

Sixteen native Spanish speakers (eight female) aged 21–29 years (mean age 24.2 years) participated in the study (one was excluded from analyses due to excessive artifacts). All were classifiable as “late bilinguals” (mean age of acquisition of French, 16.8 years, and mean years of study of French, 4.7 years). All had passed the second level of the DELF, a standardized test of French as a second language, were following a university curriculum in the French language and were living in France (mean of 5.5 months) at the time of participation. Their mean self-rating of reading expertise in the French language (on a scale from 1 to 6) was 4.0 (SD = 0.9). Another group of 15 participants were native French speakers (seven female) aged 20–24 years (mean age 21.5 years), enrolled at a French university (one participant was replaced due to excessive movement during ERP recording; 13 of these participants were the same as reported in Frenck-Mestre et al., 2008). All participants – French and Spanish – had also learned English as a second language throughout secondary school, although their fluency in this language was not tested. All participants were dominant right handed with normal or corrected-to-normal vision. Participants were paid for their participation. They all signed an informed consent form for the study, which was approved by the French ethics committee, and were fully debriefed at the end of the experiment.

#### Materials

Critical stimuli were twenty regular French verbs of the first group presented in 90 declarative present-tense sentences. Grammaticality of sentences was manipulated by verbal agreement between the subject pronoun and the verb. All six verbal persons were used. Three morphosyntactic conditions were created by manipulating the pairing of verbal person and verbal inflection, with 30 sentences per condition: correct (e.g. “je parle” 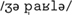), incorrect and orally realized (e.g. “je parlez” 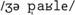), and incorrect and silent (e.g. “je parles” 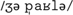). Examples are provided in **Table 1**. In the correct and phonologically realized incorrect conditions, the three singular verbal persons were seen an equal number of times (four times each) as were the 3 plural verbal persons (six times each), In the phonologically silent incorrect condition, the first person plural (“nous”) and second person singular formal/plural (“vous”) were not included as any mis-pairing gives rise to an overt phonological form; the other four forms (1st, 2nd informal and 3rd singular, 3rd plural) were seen equally. Sentences were from 5 to 10 words in length and critical verbs appeared at varying word positions, from the second to the fifth word, but never in the final position. Each verb was presented either four or five times across the 90 sentences. Forty-five additional filler sentences that did not involve morphosyntactic anomalies were included as filler sentences to distract participants’ attention from the morphosyntactic manipulation. This fillers also included correct verbal inflections of the different verbal persons in such a way that all verbal persons were seen an equal number of times in correct and incorrect conditions across the entire set of materials. A latin square design was used, such that each experimental sentence was rotated across three lists, with each occurring only once per list and in a different condition per list. Each list contained 90 experimental sentences (30 per condition) and 45 filler sentences. Each participant saw only one list and three different random orders of presentation of sentences were created per list.

#### Procedure

Participants were instructed to read sentences silently from a computer monitor while seated comfortably in an isolated room. Each trial sequence consisted of the following: a fixation cross (500 ms) followed by the stimulus sentence, which was presented visually one word at a time, each word being displayed for 450 ms followed by a 150 ms blank-screen inter-stimulus interval, followed by a “oui/non” prompt. Participants read for comprehension and made meaning-acceptability judgments at the prompt after each sentence by means of a button box.

EEG activity was recorded continuously from 21 scalp locations referenced to the left mastoid, with a sampling rate of 200 Hz. Two additional electrodes were used to monitor for horizontal and vertical eye movements. Epochs began 100 ms prior to stimulus onset and continued 1100 ms thereafter. Average ERPs were calculated off-line from trials free of artifacts (less than 3% of rejections per condition overall; no differences in rejection rate were found as a function of condition or participant group). Averaging was performed without regard to behavioral responses^1^.

#### Data analysis

Mean voltage amplitudes and peak latencies were calculated for two time windows: 400–550 and 600–800 ms. These time epochs have been associated with the N400 and/or anterior negativities and P600 components, respectively. Data acquired at midline and lateral sites were treated separately. A three-way ANOVA was performed on the mean amplitude and peak latency acquired at midline, with two levels of Group (Native French vs. Spanish–French bilinguals), and repeated measures on three levels of Verbal inflection (correct inflection, orally realized and silent inflectional errors) and Electrode (frontal, central, and parietal). Five-way ANOVAs were performed on the data acquired at lateral sites, involving Group (Native French vs. Spanish–French bilinguals), and repeated measures on three levels of Verbal inflection (correct inflection, orally realized and silent inflectional errors), two levels of Hemisphere (left, right), two levels of Site (anterior, central–parietal) and three levels of Electrode (three anterior and three central–parietal per hemisphere). The Greenhouse and Geisser (1959) correction was applied to all repeated measures with greater than one degree of freedom. All significant differences involving more than two conditions were confirmed by *post hoc* comparisons (Bonferroni).

### RESULTS

#### Behavioral data

Grammatical acceptability judgments were analyzed as a function of Group (native French vs. Spanish L1–French L2 learners) and Verbal inflection (correct, orally realized, and silent errors). There was a main effect of Verbal inflection [*F*(2,56) = 11.52, *p* < 0.01] that tended to be modified by Group [*F*(2,56) = 3.42, *p* < 0.06]. *Post hoc* comparisons revealed the main effect of Verbal inflection to be due to the difference in correct responses for silent errors compared to orally realized errors and correct inflections (*p* < 0.01). This effect was further confirmed in each group independently [French (*F*(2,28) = 10.23, *p* < 0.01) and Spanish L1–French L2 learners (*F*(2,28) = 3.39, *p* < 0.05]. Thus, the difference in the percentage of correct responses for silent errors compared to orally realized errors and correct inflections was bigger in the native French speakers in comparison to that observed in Spanish L1–French L2 learners [mean percentage of correct detections for correct sentences, orally realized and silent errors were for native French speakers 93% (SD 3.1), 96% (SD 2.9), and 72% (SD 8.1), respectively, and for Spanish L1–French L2 learners 91% (SD 3.4), 89% (SD 5.6), and 82% (6.1), respectively].

#### Event-related potentials

**FIGURE 1:**
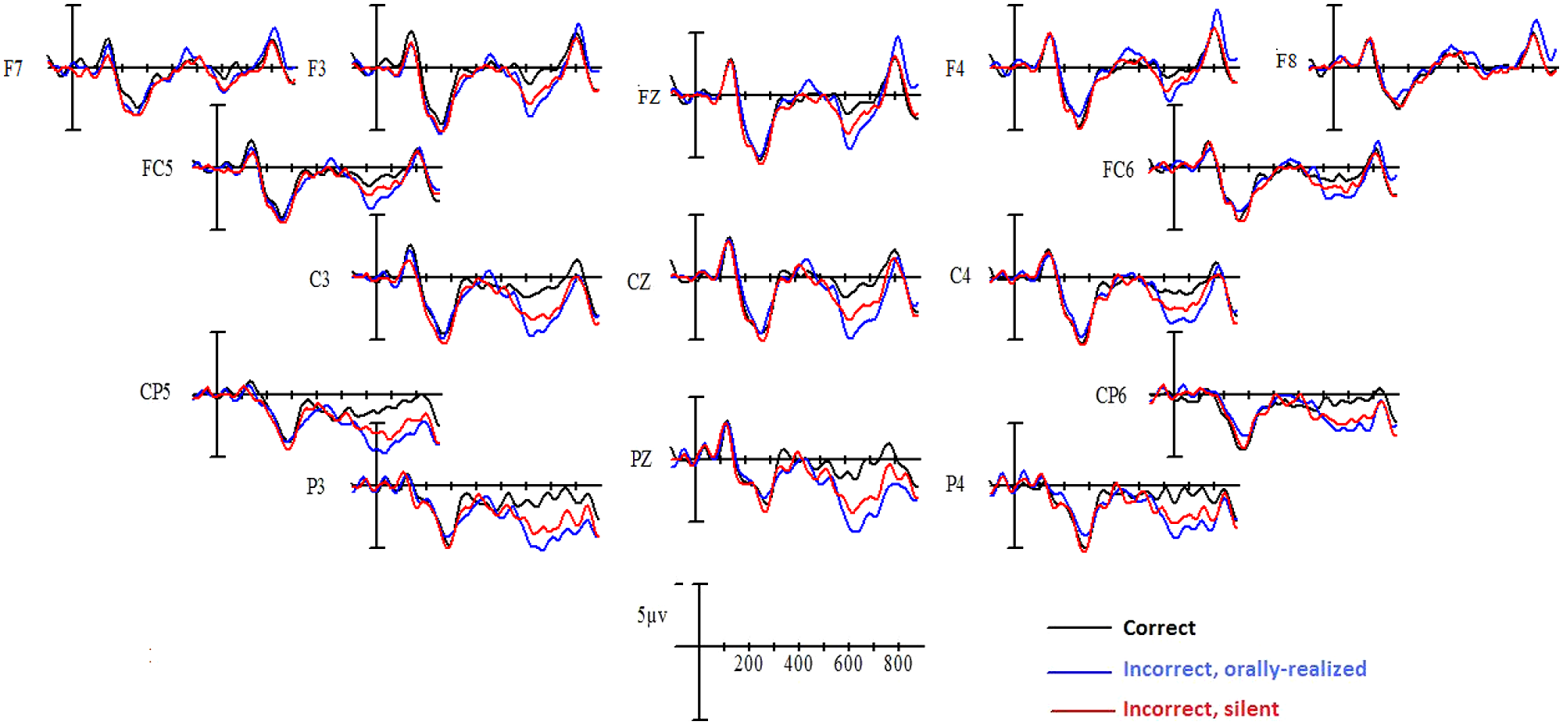
**Grand mean averages for native French speakers as a function of verbal inflection condition and electrode site**.

**FIGURE 2:**
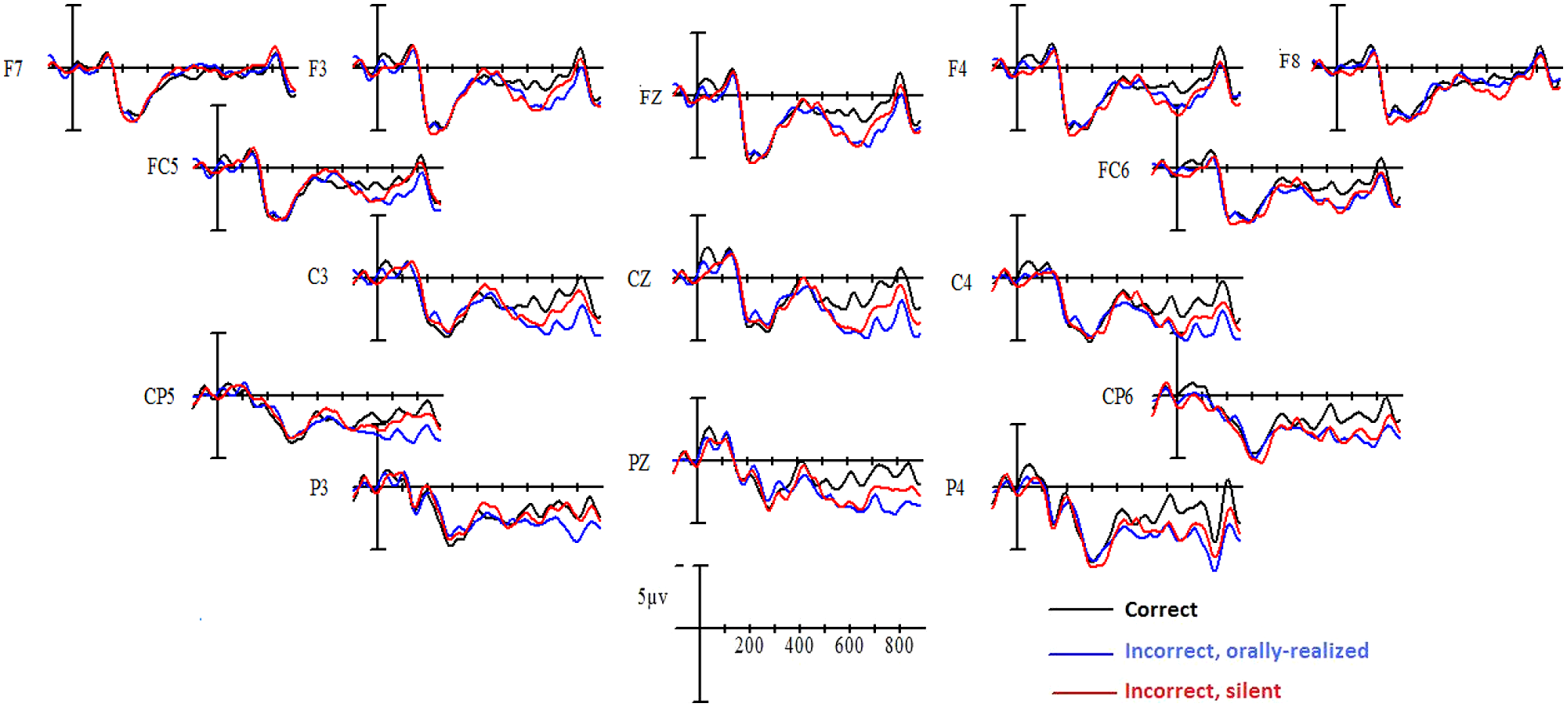
**Grand mean averages for Spanish L1–French L2 speakers as a function of verbal inflection condition and electrode site**.

Grand-average waveforms to the critical verbs for each verbal agreement condition are shown in **Figure 1** for native French and in **Figure 2** for Spanish L1–French L2 participants. As is visible in the figures, a clear “N1–P2” complex was evoked in the first 300 ms following critical word onset, for all conditions. Following this, in comparison to correctly inflected verbs, inflectional errors provoked a positive deflection that began at roughly 500 ms and persisted until around 800 ms, with a peak at roughly 600 ms, generally described as a P600 effect. This was true for both orally realized and silent errors and was observed for both native French speakers and for Spanish–French late bilinguals. In addition, a larger P600 effect was apparent for the orally realized errors compared to silent errors in both participant groups. Statistical comparisons confirmed these differences.

#### 100–300 ms epoch

Statistical analyses revealed no reliable differences across conditions in the first 300 ms following critical verb onset.

#### 400–600 ms epoch

Analysis of data in this time window revealed no effects at midline. For lateral electrodes, an effect emerged for participant Group [*F*(1,28) = 5.26, *p* < 0.02] which was modified by interactions with Hemisphere × Verbal inflection [*F*(2,56) = 3.08, *p* < 0.05]. Independent analyses performed on the data for each participant group showed this interaction to be due to the presence of a positive deflection in response to both orally realized and silent errors only for Spanish L1–French L2 learners. No such early P600 effect was apparent in the native French group for whom no reliable differences as a function of experimental factors were found in this time window.

#### 600–800 ms epoch

Analysis of data in this time window yielded a main effect of Verbal inflection [midline: *F*(2,56) = 18.41, *p* < 0.0001; lateral sites *F*(2,56) = 9.67, *p* < 0.001]. At midline, compared to well-formed sentences both orally realized and silent inflectional errors produced a significant P600 effect (*p* < 0.01 or better for both cases), which was significantly larger for orally realized errors than silent errors as confirmed by Bonferroni *post hoc* comparisons (*p* < 0.01). In addition, there was a trend for the interaction involving Verb, Group, and Electrode site [*F*(4,112) = 2.29, *p* < 0.10]. At lateral sites, there was a significant interaction between Verb, Group, and Hemisphere [*F*(2,56) = 3.83, *p* < 0.03]. Given such, independent analyses were performed on the data in this time window for each of the two participant groups.

In the native French group, an effect of Verbal inflection was observed at midline [*F*(2,28) = 7.11, *p* < 0.003] which was modified by Electrode [*F*(4,56) = 8.44, *p* < 0.001]. *Post hoc* comparisons revealed that compared to grammatically correct cases, orally realized errors elicited a significant P600 response at all three midline electrodes (*p* < 0.01 or better) whereas silent errors produced a reliable P600 response at central (*p* < 0.001) and parietal (*p* < 0.001) but not at frontal sites. In addition, orally realized errors produced a significantly larger P600 effect than silent errors at central (*p* < 0.03) and parietal sites (*p* < 0.001). The effect of Verbal inflection was also significant at lateral sites [*F*(2,28) = 3.91], revealing a significant P600 effect for both orally realized and silent errors. The P600 effect was present over central–parietal but not over anterior sites and it varied as a function of Verbal inflection [*F*(2,28) = 8.57, *p* < 0.004]. *Post hoc* comparisons revealed no P600 effect at anterior lateral sites, whereas at central–parietal lateral sites both types of error produced a robust P600 effect. In addition, this P600 effect was larger for orally realized errors (*p* < 0.0001) than for silent errors (*p* < 0.001), although the direct comparison of the two error conditions at posterior lateral sites did not show a reliable difference.

In the Spanish L1–French L2 learners group, a significant effect of Verbal inflection was observed at midline [*F*(2,28) = 13.8, *p* < 0.0001], which did not interact with Electrode. *Post hoc* comparisons at midline sites confirmed that compared to control sentences, orally realized and silent inflectional errors both produced a P600 effect (*p* < 0.01 or better) and differed from each other at all three midline sites (*p* < 0.05). At lateral sites, there was also an effect of Verbal inflection [*F*(2,28) = 7.87, *p* < 0.01], which tended to be modified by Hemisphere [*F*(2,28) = 3.37, *p* < 0.06] and was significantly so by the interaction involving Verbal inflection, Site (anterior/posterior), and Electrode [*F*(4,56) = 10.45, *p* < 0.001]. At anterior lateral sites, *post hoc* comparisons revealed that orally realized errors produced a more widespread P600 effect over the left than right hemispheres whereas the effect for silent errors was only significant over the right hemisphere (*p* < 0.03). At posterior lateral sites, an effect of Verbal inflection was observed [*F*(2,28) = 10.83, *p* < 0.01], with orally realized errors producing a robust widespread P600 effect (*p* < 0.001) and silent errors producing a more reduced effect (*p* < 0.05) that was also significantly smaller than that produced by orally realized errors over some electrodes (*p* < 0.002).

### DISCUSSION

The results of Experiment 1 clearly demonstrate online sensitivity to morphosyntactic violations for Spanish L1–French L2 learners, in like manner to native French speakers, as revealed by a P600 response to these violations. Of principal interest in the present study was whether phonological cues would enhance the processing of morphosyntactic violations during silent reading. For native speakers, orally realized errors produced a greater P600 effect than silent errors, thus confirming previous results (Frenck-Mestre et al., 2008). In the same way, Spanish L1–French L2 learners showed a graded sensitivity to verbal inflection violations as a function of the presence of phonological cues. Indeed, the P600 response to orally realized errors was significantly larger than that observed to silent errors. The differentiated ERP response in non-native speakers contrasts nonetheless with recent work by McLaughlin et al. (2010). Using the same materials as in the present study but with less advanced English learners of French, these authors reported differences in sensitivity to verbal person violations as a function of whether these violations were orally realized or silent, but only on grammaticality judgments, not in the ERP response to these violations as reported here. In line with McLaughlin et al. and in view of our own results, we can forward the hypothesis that in less advanced L2 learners online processing may not be rapid and/or systematic enough to show a robust effect of phonological realization during reading in the ERP trace. We will re-examine this question in the general discussion in light of the results of Experiment 2.

To further investigate the role of phonology during silent reading and how such may impact grammatical processing as a function of L2 experience, we conducted a second experiment in which we again manipulated phonological variation. In line with interactive models of phonology and orthography during reading (Harm and Seidenberg, 2004) it is plausible that readers benefited from all cues available to them, whether phonological or orthographic. It is possible that the inclusion of plural pronouns in our first experiment may have enhanced the effect of oral realization that we observed, due to differences in orthography. Indeed, for the three singular pronouns the orthographic overlap between correct inflections and both the orally realized and silent violations was identical (one letter different in each case). Such was not the case for the plural, for which silent errors had more letters in common with correct inflections than orally realized errors did (for a more indepth discussion, see Frenck-Mestre et al., 2010). To address this question and to ascertain that the differences in processing we observed were indeed due to the oral realization of morphology, we conducted a further experiment in which orthographic cues were reduced. This was done by restricting the verbal person manipulation to the three singular pronouns (1st, 2nd, and 3rd person). In said case, it is possible to hold orthographic variations constant meanwhile varying phonological overlap.

## EXPERIMENT 2

In Experiment 1, we manipulated the presence vs. absence of oral cues to verbal agreement by mispairing inflection for both singular and plural pronouns. Our results clearly revealed an effect of phonological realization on agreement processing for both participant groups. The goal of Experiment 2 was to determine whether the effect of phonological realization observed in Experiment 1 was principally driven by phonological rather than orthographic cues. If so, we would expect this effect to persist when the amount of orthographic mismatch was identical across incorrect agreement conditions. Therefore, we used the three singular pronouns (“je,” “tu,” “il/elle”), for which verbal inflections vary by one letter and mispairing inflections may result in either orally realized or silent errors (see **Table 2**). We can predict that the presence of oral cues should enhance the reader’s capacity to detect these errors. Based on previous ERP studies of written materials, we expected a variation in the amplitude of P600 and/or early negativities to verbal agreement errors as a function of oral cues, for native French and Spanish L1–French L2 speakers. It is also possible that an N400 effect could be elicited by morphological mispairings, especially in the L2 group. Indeed, various studies of adult L2 learners have reported N400 effects to just such errors, although none have manipulated the presence of overt oral cues to morphological variation (McLaughlin et al., 2010; Foucart and Frenck-Mestre, 2012; Morgan-Short et al., 2012; Tanner et al., 2013, 2014). The group of Spanish–French learners was, akin to the group tested in Experiment 1, immersed in French at the time of testing. All L2 participants had also had several years of formal study of the French language. As such, this group was comparable to that tested in the first experiment and considerably more advanced than the L2 learners tested by McLaughlin et al. (2010). Again, if L2 proficiency, as determined by either formal learning or amount of exposure is crucial to using phonological cues during reading in general and syntactic processing in particular, then we can predict similar results as found in Experiment 1, provided that the phonological cues are sufficient to produce effects in the absence of additional orthographic cues.

**Table 2:** Examples of the three sentence conditions (correct, incorrect and orally realized, incorrect and silent) for the three singular verbal persons in French used in Experiment 2.

Sentence onset	Correct	Incorrect, phonologically realized	Incorrect, phonologically silent	Sentence end
Le soir	je regarde tu regardes il/elle regarde	regardez regardez regardez	regardes regarde regardes	des films

### METHOD

#### Participants

Fifteen native French speakers (eight female) aged 18–25 years (mean age 20 years) and 15 native Spanish speakers (nine female) aged 21–29 (mean age 26.8 years) participated in this study. None of the participants had taken part in the first experiment. All subjects were dominant right handed with normal or corrected-to-normal vision. They were paid for their participation and signed an informed consent form for the study, which was approved by the French ethics committee. They were fully debriefed at the end of the experiment. Spanish speakers were classifiable as “late bilinguals” (mean age of acquisition of French, 23.6 years, and mean years of study of French, 3.5 years). All had passed the second level of the DELF, a standardized test of French as a second language, were following a university curriculum in the French language and were living in France (mean of 6 months) at the time of participation. Their mean self-rating of reading expertise in the French language (on a scale from 1 to 6) was 4.2 (SD = 0.77). All participants – French and Spanish – had also learned English as a second language throughout secondary school, although their fluency in this language was not tested.

#### Materials

An entirely new set of sentences was created for the purpose of this experiment. Critical stimuli were 30 regular French verbs from the first group (20 taken from Experiment 1) which were presented in 90 declarative present-tense sentences. Grammaticality of sentences was manipulated by verbal agreement between the subject pronoun and the verb. As in Experiment 1, 3 morphosyntactic conditions were created by manipulating the pairing of verbal person and verbal inflection, with 30 sentences per condition: correct (e.g. “je regarde”), incorrect and orally realized (e.g. “je regardez”), and incorrect and silent (e.g. “je regardes”). In contrast to Experiment 1, only the 3 singular persons were included. Sentences were from 5 to 10 words in length and critical verbs appeared at varying word positions, from the second to the fifth word, but never in the final position. Three lists were created such that each experimental sentence was rotated across lists in a Latin square design, with each occurring only once per list and in a different condition per list. In each condition, the three singular pronouns were seen an equal number of times (10 per each, with five masculine and five feminine for the third person singular). Sixty additional filler sentences that did not involve morphosyntactic anomalies were included to distract participants’ attention from the syntactic manipulation. These fillers also provided a balance for correct and mispaired verbal inflections across the entire set of materials. Each participant saw only one list and three different random orders of presentation of sentences were created per list.

#### Procedure

The procedure was identical to that of Experiment 1.

#### Data analysis

This was identical to that of Experimental 1 with the exception that the time epoch associated with the N400 and/or anterior negativity was examined in the 300–500 ms time window, and that associated with the P600 component was shifted to the 500–700 ms and 700–900 time windows based on visual inspection of waveforms.

### RESULTS

#### Behavioral data

Behavioral responses were analyzed based on grammatical acceptability judgments. A repeated-measures ANOVA involving Group (Native French vs. Spanish–French bilinguals) as a between subjects factor and Verbal inflection (correct, silent, and orally realized errors) as a repeated measure revealed a main effect of Verbal inflection [*F*(1,43) = 27.42, *p* < 0.01] which did not interact with Group (*F* < 1). Orally realized errors were better detected than silent errors and this was true for both participant groups [mean percentage of correct detections for correct sentences, orally realized and silent errors were 94% (SD 2.3), 92% (SD 3.5), and 73% (SD 8.2) for native French speakers and 89% (SD 4.7), 86% (SD 6.4), and 61% (SD 15.9) for Spanish L1–French L2 learners, respectively]. Given the differences across conditions as concerns the detection of errors, ERPs were calculated independent of responses. Moreover, as shown by numerous studies, behavioral responses are not necessarily indicative of cortical sensitivity (McLaughlin et al., 2004; Foucart and Frenck-Mestre, 2012; but see Foucart and Frenck-Mestre, 2011).

#### Event-related potentials

**FIGURE 3:**
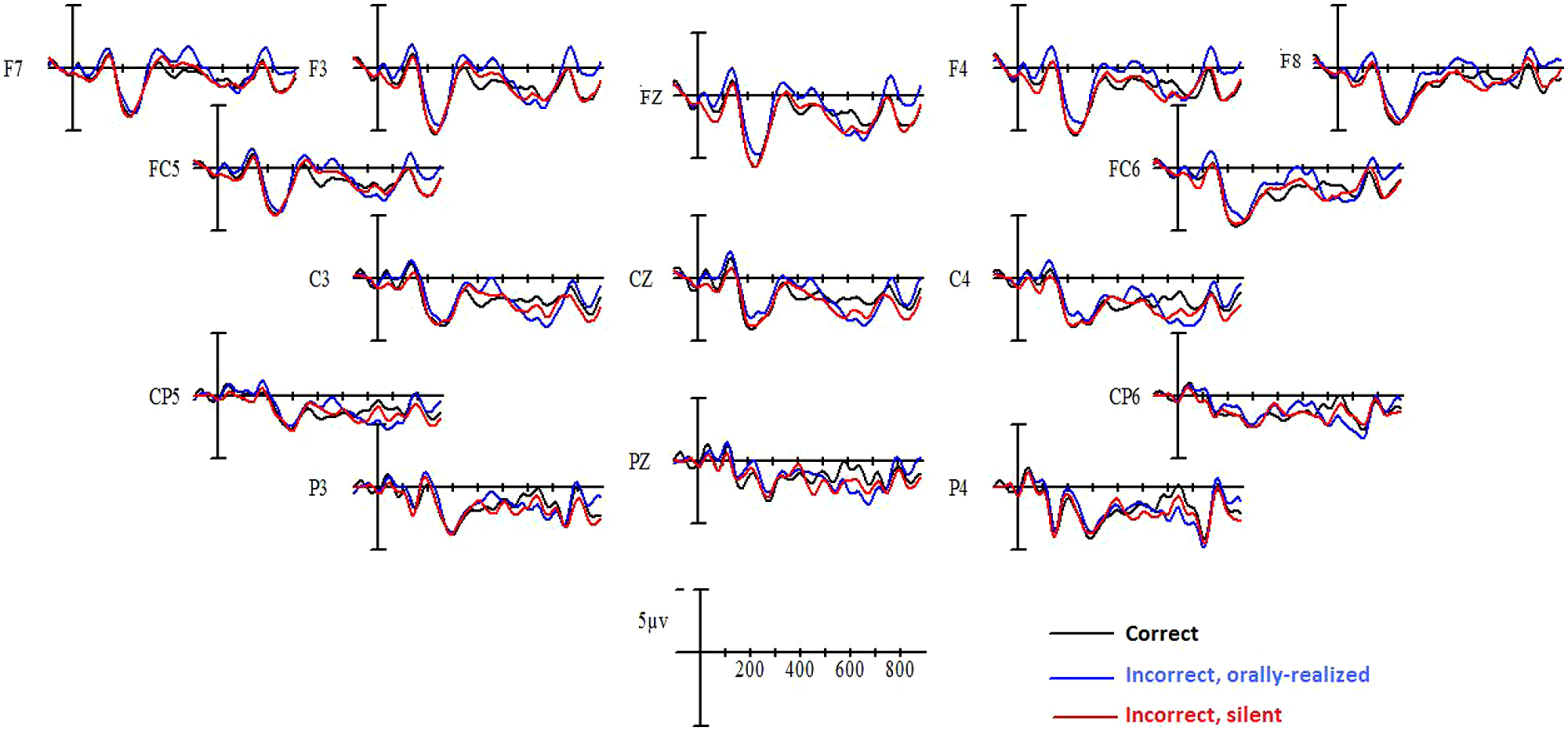
**Grand mean averages for native French speakers as a function of verbal inflection condition and electrode site**.

**FIGURE 4:**
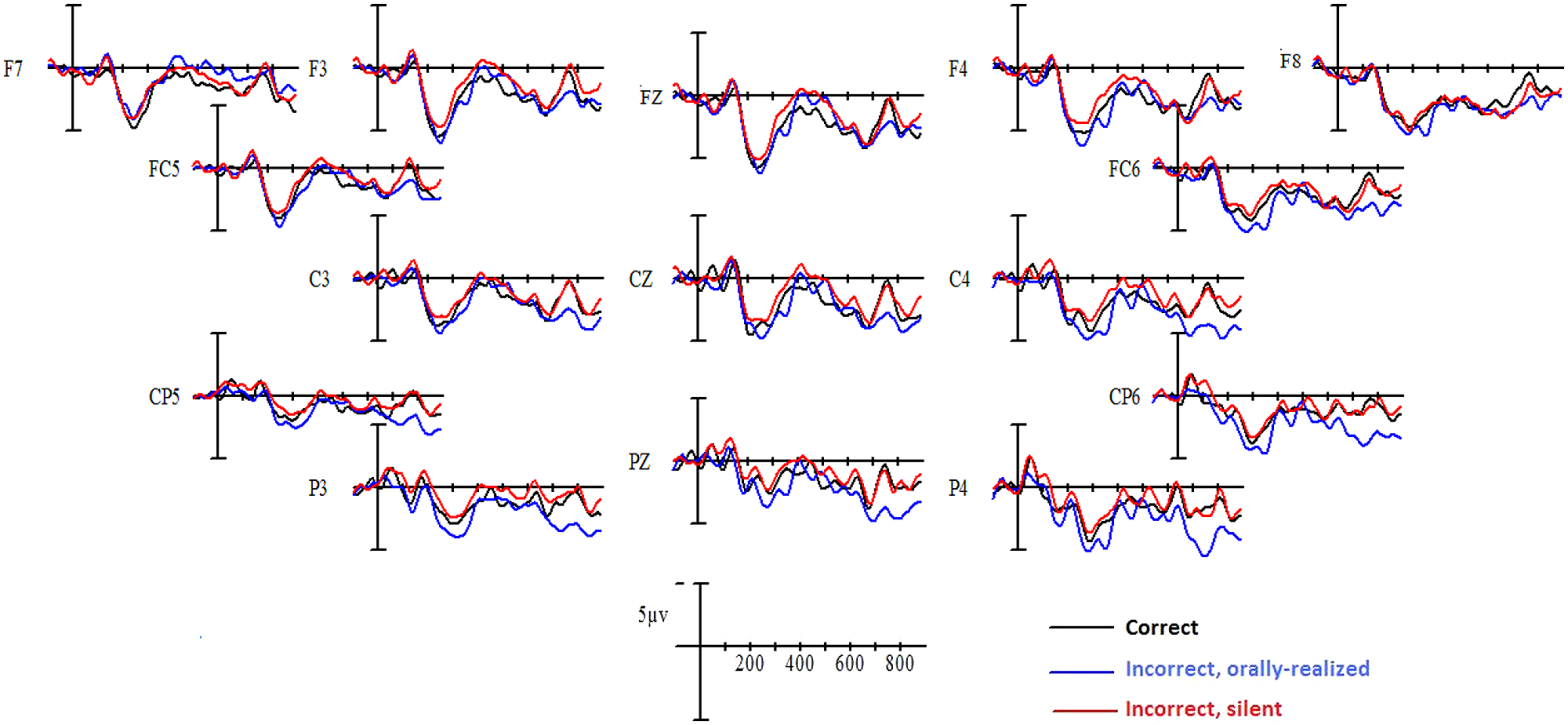
**Grand mean averages for Spanish L1–French L2 speakers as a function of verbal inflection condition and electrode site**.

Grand-average ERPs elicited by critical verbs in the three verbal agreement conditions (correctly inflected verbs, orally realized, and silent errors) are shown in **Figure 3** for native French and in **Figure 4** for Spanish L1–French L2 learners. Visual inspection of these waveforms revealed a clear “N1–P2” complex evoked in the first 300 ms following critical word onset, for all conditions. After 300 ms, two effects emerged. First, a negative component was observed over the left and right hemispheres, beginning around 300 ms and persisting until 500 ms, for inflectional errors as compared to correct verbal agreement. This negativity was observed predominantly in the native French participant group and mainly for orally realized inflectional errors. Following this negativity, inflectional errors provoked a positive deflection in comparison to correctly inflected verbs. This positivity presented different onset latencies across participant groups. For native French speakers positivity began at approximately 500 ms and persisted until roughly 800 ms, whereas for Spanish L1–French L2 learners the positivity began at 700 ms and persisted beyond 800 ms. Thus the P600 effect was delayed in L2 learners. ANOVAs performed on the mean amplitude data confirmed these effects.

#### 300–500 ms epoch

Statistical analyses in this time window yielded a significant Verbal inflection by Group interaction at both midline [*F*(2,56) = 3.79, *p* < 0.03] and lateral sites [*F*(2,56) = 3.70, *p* < 0.03]. Independent analyses were subsequently performed on the data for each participant group. For native French speakers, a main effect of Verbal inflection was observed only at lateral sites [*F*(2,28) = 4.06, *p* < 0.04]. Planned comparisons confirmed reliable differences between correctly inflected verbs and orally realized errors at lateral sites (*p* < 0.03) while no differences were observed between correctly inflected verbs and silent errors (*p* > 0.15). The comparison of orally realized and silent errors showed a reliable difference (*p* < 0.03). For Spanish L1–French L2 learners, the main effect of Verbal inflection approached significance only at midline [*F*(2,28) = 2.97, *p* < 0.08]. *Post hoc* comparisons revealed that silent inflectional errors were more negative compared to correctly inflected verbs (*p* < 0.02). No differences between orally realized errors and correctly inflected verbs or between the two error conditions were found in this time window for this group.

#### 500–700 ms epoch

Analyses of the data in this time window showed a significant interaction between Group and Verbal inflection at midline [*F*(2,56) = 4.36, *p* < 0.01], while at lateral sites Verbal inflection interacted with Group and Site [*F*(2,56) = 2.65, *p* < 0.03]. Separate analyses were subsequently performed on the data for each participant group. For native French speakers, a main effect of Verbal inflection was observed at midline [*F*(2,28) = 4.35, *p* < 0.02] and at lateral sites [*F*(2,28) = 4.06, *p* < 0.02]. *Post hoc* comparisons (Bonferroni) at midline showed that both orally realized and silent errors differed from correct sentences (*p* < 0.01), whereas orally realized and silent errors did not differ from each other. At lateral sites, the effect of Verbal inflection was modified by Site [*F*(2,28) = 4.15, *p* < 0.05]. Separate analyses at anterior and posterior sites revealed a main effect of Verbal inflection only at posterior sites [*F*(2,28) = 4.25, *p* < 0.03]. At posterior sites, *post hoc* comparisons confirmed reliable differences between correctly inflected verbs and orally realized errors (*p* < 0.03), while no differences were found between correctly inflected verbs and silent errors (*p* > 0.15). The direct comparison of the two error conditions showed a small trend (*p* < 0.11).

Analyses conducted on Spanish L1–French L2 speakers in this time window showed no effect of Verbal inflection or significant interactions at any electrode site (*F* < 1).

#### 700–900 ms epoch

Analyses of data in this time window yielded a significant interaction between Verbal inflection and Group at midline [*F*(2,56) = 6.27, *p* < 0.01] and at lateral sites [*F*(2,56) = 5.35, *p* < 0.01]. Separate ANOVAs were subsequently conducted on the data for each participant Group. Native French speakers showed no significant effects or interactions at any electrode site (*F* < 1). In contrast, Spanish L1–French L2 speakers revealed a significant effect of Verbal inflection at midline [*F*(2,28) = 5.93, *p* < 0.01] which was modified by Electrode site [*F*(4,56) = 3.73, *p* < 0.03]. At midline, *post hoc* comparisons revealed that compared to correct cases, orally realized errors elicited a significant P600 response at central (*p* < 0.02) and parietal (*p* < 0.001) sites, while no significant effect was observed at any electrode sites for silent errors, for any comparison. At lateral sites, a significant effect of Verbal inflection was observed [*F*(2,28) = 4.27, *p* < 0.02] which interacted with Site [*F*(2,28) = 9.6, *p* < 0.01]. *Post hoc* comparisons at anterior and posterior sites revealed that at posterior sites, orally realized but not silent errors elicited a significant P600 (*p* < 0.01) in comparison to correct sentences, whereas at anterior sites no reliable P600 response was observed. Further, orally realized errors produced a significant P600 effect compared to silent errors (*p* < 0.01).

### DISCUSSION

The goal of Experiment 2 was to confirm that the effect of phonological realization observed in Experiment 1 could be attributed to the additional presence of phonological cues rather than to orthographic cues alone. The results, obtained under conditions for which the amount of orthographic mismatch across verbal agreement conditions was controlled, showed again that orally realized verbal person violations provoked a reliable P600 response in comparison to correct cases and this was true for both native and non-native participants. In addition, compared to silent errors orally realized violations elicited a greater early negativity in native French speakers. Given its distribution, this effect can best be characterized as a member of the broad class of early negativities which has been reported in various studies (Rodriguez-Fornells et al., 2001; Hahne et al., 2006; Morgan-Short et al., 2012). In contrast to orally realized inflectional errors, silent errors elicited only a small effect. In the group of native French participants, silent errors only produced a significant P600 response at midline sites. For Spanish L1–French L2 speakers, the difference between orally realized and silent inflectional errors was clear and widespread; while orally realized errors elicited a P600, albeit in a late time window, silent errors did not produce any reliable effect in the ERP waveform.

## GENERAL DISCUSSION

The present set of studies showed that phonological cues enhance the processing of inflectional morphology when reading silently in either one’s first or second language. Specifically, the processing of verbal inflectional errors produced a larger P600 effect when violations involved both orthographic and phonological cues relative to when only inaudible morphological cues are available. These results provide evidence of the contribution of phonology to morphological processing in both native and non-native readers. The general findings here are consistent with those of previous studies that show an impact of phonological cues on the processing of inflectional morphology in French (Frenck-Mestre et al., 2008, 2010; Carrasco and Frenck-Mestre, 2009; McLaughlin et al., 2010) and confirm a systematic benefit from the presence of phonological cues under conditions where orthographic overlap across experimental conditions was held constant (Experiment 2). Although this was true for both native French speakers and Spanish L1–French L2 speakers, distinct neural responses were observed for each group as a function of the presence vs. absence of phonological cues when processing morphosyntactic markers.

For native speakers, a bilateral early negativity was evoked in response to orally realized errors in comparison to correctly inflected verbs. This was found, however, only when minimal orthographic differences between correct and orally realized errors were present (i.e., only in Experiment 2). In contrast, silent errors did not elicit any significant negativity, under any conditions. Both in terms of timing and distribution, the anterior negativity observed for orally realized errors fall within the range of variations that have been reported in previous studies for syntactic violations (Rodriguez-Fornells et al., 2001; Hahne et al., 2006; cf. Molinaro et al., 2011 for a review). The nature of this early negativity has been associated with a fast and automatic syntactic analyzer involving an initial detection of the grammatical error (Friederici, 1995, 2002; Hahne and Friederici, 1999). In line with this assumption, the fact that this negativity was present for orally realized but not for silent errors would suggest that phonological information has an effect on the first morphosyntactic analysis allowing a fast detection of orally realized inflectional errors. However, the interpretation of this early negativity should be considered with caution due to its lack of consistency across Experiments 1 and 2 and other previous studies (Frenck-Mestre et al., 2008, 2010; McLaughlin et al., 2010; Steinhauer, 2014; Tanner, 2014). Indeed, there is still considerable debate as to the significance and very nature of early negativities associated with syntactic processing in the monolingual literature (Osterhout, 1997; Molinaro et al., 2011; Tanner, 2014) which has seen repercussions in the literature on L2 processing (Frenck-Mestre, 2005; Steinhauer et al., 2009; Foucart and Frenck-Mestre, 2012; Steinhauer, 2014).

The results for native speakers showed that compared to orally realized errors, silent errors produced a smaller P600 effect in Experiment 1 and a reduced distribution in Experiment 2. The ERP differences observed for these two types of errors suggest that the presence of oral cues enhanced the syntactic analysis/reanalysis of violations of inflectional morphology in French. These results are in line with those obtained in previous off-line studies for native speakers of French (Negro and Chanquoy, 2000; Largy and Fayol, 2001) where phonologically realized morphemes induced fewer inflectional errors for verbal and nominal agreement in a written production task. It is noteworthy, nonetheless, that the difference observed between orally realized and silent inflectional errors was more pronounced in Experiment 1 in which the extent of orthographic differences between correctly inflected and erroneous cases was larger for orally realized than for silent errors. As such, the present results support the hypothesis that the added presence of orthographic cues indeed enhanced the effect of the oral realization of errors.

For the Spanish L1–French L2 speakers, no reliable early negativity was observed in response to verbal agreement errors. This result could fit, in a first instance, with the assumption that early negativities are restricted to native processing (Hahne, 2001) and or that they are associated with more advanced levels of processing (Steinhauer et al., 2009). However, the fact that various studies involving native speakers do not report any LAN effects in response to syntactic violations (Hagoort et al., 1993; Osterhout and Mobley, 1995; Osterhout et al., 2002; Frenck-Mestre et al., 2008; Foucart and Frenck-Mestre, 2011, 2012) renders difficult the interpretation of this absence of significant negativity effects in non-native speakers as a non “nativelike” processing. Indeed, the inconsistent presence of these LAN effects in native brain responses requires further research to reveal the underlying cognitive processes and the antecedent conditions that elicit or modulate it (Osterhout, 1997; Molinaro et al., 2011; Steinhauer, 2014; Tanner, 2014). Moreover, it has recently been suggested that individual differences in native speakers can account for the presence versus absence of early negativities to syntactic manipulations (Tanner, 2014). Clearly, further work is in order to clarify this issue.

In the present study, L2 learners showed a larger P600 effect when processing orally realized errors, as compared to silent errors and correctly inflected verbs. These results contrast with those reported in McLaughlin et al. (2010) where L2 learners showed no ERP difference between orally realized and silent errors. The greater on-line capacity to detect orally realized errors observed in the present study might be associated with the relative high language proficiency in our L2 learners. Increased second language processing might enable the activation of phonological information, which could have enhanced the L2 learner’s capacity to detect morphological violations involving phonological cues. In addition, the results of Experiment 2 replicate those reported in Frenck-Mestre et al. (2008), showing an absence of an ERP response to silent errors for L2 learners. One interpretation for these results is that non-native participants in Experiment 2 were not systematically sensitive to morphological errors that are not overtly realized. It is, therefore, possible that the L2 participants’ response to silent inflectional errors was not strong enough to elicit a visible ERP response in the second experiment in contrast to the results of Experiment 1. This inherent heterogeneity of response in the L2 participants may have contributed to the absence of an effect for silent inflectional errors. Indeed, behavioral data in Experiment 2 showed that orally realized errors were detected significantly better than silent errors.

As outlined above, the effect of the phonological realization of morphology differed across experiments. Indeed, the difference in the ERP response to orally realized and silent errors was larger and more widespread for both participant groups in Experiment 1 than in Experiment 2. One possible explanation is that more robust effects are observed for experimental conditions in which the number of orthographic cues present in verbal inflection errors is greater. In Experiment 1, orally realized errors included all six verbal persons producing, therefore, orthographically more salient errors compared to Experiment 2 in which only the three singular persons were used (e.g., “nous_1st,plural_ parlent*_3rd,plural_/parlons_1st,plural_” compared to (“je_1st,sing_ parlez*_2nd,singformal/plural_/parle_1st,sing_”). This relative orthographic advantage may have improved the processing of verbal inflection errors in Experiment 1. This finding is in line with assumption that readers benefit from all linguistic input when processing language (Brysbaert et al., 2000; Harm and Seidenberg, 2004; Frenck-Mestre et al., 2010). Under a connectionist framework, a mutual dependence of orthographic and phonological codes operates in the computation of a written word (Harm and Seidenberg, 2004). Thus, the different contribution of orthographic and phonological codes across experiments may have impacted the processing of words.

An important issue for this paper was to confirm the contribution of phonological cues to the online processing of inflectional morphology. In line with Brysbaert et al. (2000), the results obtained in the present study suggest that orthographic cues that are not phonologically represented in inflectional morphology can still be processed, though perhaps in a more effortful way. Our results also suggest that the presence of phonological cues can enhance the processing of inflectional morphology, even under conditions for which the amount of orthographic mismatch was identical. Furthermore, the present study provides important evidence relative to both native and non-native speakers’ use of these cues during silent reading.

Finally, the impact of phonological cues on morphological variations is not limited to verbal processing. Indeed, the processing of other morphological inflections such nominal gender concord has been found to be enhanced by the presence of phonological cues (Carrasco and Frenck-Mestre, 2009; Foucart and Frenck-Mestre, 2011, 2012; for a review see Frenck-Mestre et al., 2010). In addition, this effect of phonology has been observed in both native and non-native speakers with diverse language backgrounds. Thus, the current set of experiments points to an active use of phonological information when reading silently and such is true, moreover in both first and second language processing.

## Conflict of Interest Statement

The authors declare that the research was conducted in the absence of any commercial or financial relationships that could be construed as a potential conflict of interest.

## Footnotes

1 In both experiments, ERP data were analyzed based on all trials that were not contaminated by artifact. Response-contingent analyses are problematic in the present study given the unequal number of trials across experimental conditions. Indeed, in the phonologically silent condition, there was a substantial percentage of trials on which participants, whether native or L2 speakers, failed to consciously report an error, which was not the case for sentences in the orally realized condition. We nonetheless looked at the ERP data based on response-contingency. The results reported herein held up for all comparisons between orally realized inflectional errors and correct inflections. The results for the silent inflectional errors were less robust. Nonetheless, since excluding those trials in which the behavioral response was incorrect led to excluding more than 40% of the data, the reliability of these analyses is questionable.
